# Prevalence and Treatment Outcomes of Arrhythmias in Patients with Single Ventricle Physiology over the Age of 40 Years

**DOI:** 10.3390/jcm11216568

**Published:** 2022-11-05

**Authors:** Claudia Pujol, Gabriele Hessling, Marta Telishevska, Sandra Schiele, Isabel Deisenhofer, Peter Ewert, Oktay Tutarel

**Affiliations:** 1 Department of Congenital Heart Disease and Paediatric Cardiology, German Heart Centre Munich, TUM School of Medicine, Technical University of Munich, 80636 Munich, Germany; 2 Department of Electrophysiology, German Heart Centre Munich, TUM School of Medicine, Technical University of Munich, 80636 Munich, Germany; 3 DZHK (German Centre for Cardiovascular Research), Partner Site Munich Heart Alliance, 80992 Munich, Germany

**Keywords:** Fontan, single-ventricle, arrhythmias

## Abstract

Background: Arrhythmias are a well known complication in patients with single ventricle physiology (SVP). However, there is still a lack of data regarding arrhythmias in older patients. The aim of this study was to analyze arrhythmia type and frequency, treatment and recurrence rates in patients with SVP over the age of 40 years. Methods: Data was obtained retrospectively from clinical records. All patients > 40 years with SVP with arrhythmias between 2005 and 2018 were included in the study. Treatment was classified as medical, interventional (electrophysiological studies (EPS) in combination with catheter ablation) or direct current cardioversion (DCCV). Results: Altogether, 29 patients (11 female; mean 47.5 ± 4.6 years) with 85 arrhythmia episodes were identified. The median follow-up time was 6.3 years. Cavo-tricuspid (CTI) and non-CTI related intra-atrial reentrant tachycardia (IART) and atrial fibrillation (AF) were most common (48.2% and 37.6%, respectively). In total, 18 EPS/ablations were performed in 9 patients and 52 DCCVs in 20 patients. Acute success was 98% for DCCV and 72.2% for EPS/ablation. Recurrence rate was high (70% for DCCV and 55% for EPS). AT recurrences occurred after a median of 8 and 2.5 months, respectively. On multivariate analyses, age was the only risk factor for arrhythmia recurrence (HR 0.58, 95% C.I. 0.43–0.78, *p* < 0.0001). Pacemaker implantation was necessary in seven patients (AV block *n* = 4, sinus node dysfunction *n* = 3) and one patient received an ICD for secondary prophylaxis. Sudden death occurred in three patients. Conclusions: The most common arrhythmias in patients with SVP > 40 years are IART and AF. Arrhythmia recurrence following EPS or DCCV is frequent. Older age is an independent risk factor for arrhythmia recurrence.

## 1. Introduction

Some of the most complex congenital heart defects (CHD) result in a single-ventricle physiology (SVP). Since the introduction of the Fontan circulation in 1968, the prognosis of these patients has radically improved [1]. However, patients are prone to cardiac complications and sequelae that often necessitate medical, interventional or even surgical therapy [1,2]. 

One of the most common complications is the development of arrhythmias [2,3]. Arrhythmias occur in every type of SVP and after all kinds of surgical palliation, although the most recently used techniques have reduced its incidence [1]. Atrial tachycardia (AT) are present in up to 60–80% of patients and sinus node dysfunction in 30–63% of patients with atrio-pulmonary connection [1]. Arrhythmias have a prognostic implication and are associated with an increased morbidity and mortality in SVP patients [1]. Moreover, ATs in SVP are frequently resistant to antiarrhythmic drug therapy [2]. Time of follow-up, atrio-pulmonary shunt, older age at Fontan operation, preoperative and early postoperative tachycardia, and moderate to severe atrioventricular valve regurgitation have been identified as risk factors for the appearance of AT [1]. 

There is vast literature regarding arrhythmias in SVP at a younger age. However, there is still a lack of information regarding arrhythmias in older patients. We previously reported the clinical course of patients with SVP > 40 years, of whom almost 60% needed specific treatment for arrhythmic complications [3].

The aim of this study is to analyze the type and frequency of arrhythmias in SVP patients > 40 years as well as treatment strategies, success rates and risk factors for recurrence.

## 2. Materials and Methods

In this single-center study, all patients with a diagnosis of SVP ≥ 40 years of age or turning 40 years at any point between January 2005 and March 2018 (as previously described [3]) were included. The time-point of inclusion and begin of follow-up was either the date of their 40th birthday during this period or, if the patient was already 40 years old in the year 2005, the first visit after the 1st of January 2005. Patients who developed arrhythmias after the age of 40 years were compared to those without arrhythmias. 

Demographic data and information on medical/surgical history were retrieved from hospital records. Type of CHD (double inlet left ventricle-DILV, tricuspid atresia- TA or miscellaneous) and type of surgical therapy (atrio-pulmonary connection, total cavo-pulmonary connection-TCPC, conversion to TCPC) was registered. Functional class according to the New York Heart Association (NYHA) classification and ventricular function assessed by echocardiography were included in the analysis. Due to the heterogeneity of cardiac anatomy, a qualitative, subjective assessment of systemic ventricular function from multiple two-dimensional echocardiography was used to classify it as normal, mildly, moderately or severely impaired as described previously [4]. Mortality as well as cause of death during follow-up were also reported. 

Atrial tachycardias were classified as cavo-tricuspid (CTI) and non-CTI related intra-atrial reentry tachycardia (IART), atrial fibrillation (AF) or focal atrial tachycardia (FAT). Ventricular arrhythmias comprised sustained ventricular tachycardia (VT) and ventricular fibrillation (VF). Bradyarrhythmias such as acquired symptomatic sinus node dysfunction, defined as the disturbances in impulse generation of the sinus node and propagation [5], encompassing sinus bradycardia, sinoatrial block, chronotropic incompetence sinus arrest and bradycardia-tachycardia syndrome [6], and higher degree atrioventricular-block (AV-block) were also documented. Frequency and type of treatment as well as success rates were analyzed. Treatment was classified as pharmacologic treatment (amiodarone), electrophysiology study with ablation (EPS), DCCV, or pacemaker/internal cardioverter defibrillator implantation (ICD). Acute success was defined as conversion to sinus rhythm by direct current cardioversion (DCCV). During EPS, acute success was defined as tachycardia termination and non-inducibility, confirmation of bidirectional conduction block (if applicable) or confirmation of pulmonary vein isolation. 

In Fontan patients, pulmonary artery pressure will not reach the threshold used to define pulmonary hypertension in patients with a biventricular circulation; hence pulmonary hypertensive vascular disease was defined according to current recommendations for Fontan patients taking into account a mean transpulmonary gradient > 6 mmHg or pulmonary vascular resistance index > 3 WU × m^2^ [7,8].

The primary endpoint was recurrence of any arrhythmia requiring treatment. 

Statistical analyses were performed using SPSS version 25 (IBM Corp, Armonk, NY, USA). Continuous variables are presented as mean ± standard deviation (SD) or median (interquartile range-IQR), whereas categorical variables are presented as number (percentage). Comparisons between groups were performed using the Mann–Whitney U test or Student’s *t*-test for continuous and Chi-square test for categorical variables. Univariate Cox proportional hazards analysis was used to assess the association between variables and the primary endpoint. Significant variables (*p* < 0.05) were subsequently included in a multivariable Cox proportional hazards analysis model. All tests were performed two-sided and for all analyses, a *p*-value <0.05 was considered statistically significant.

## 3. Results

Among 49 patients described in our first paper [3], we identified 29 patients (59.2%) with arrhythmias after the age of 40 years (mean age 47.5 ± 4.6 years, 37.9% female). Median follow-up time was 6.3 years (IQR 3.7–9.3). 

Comparing both groups, with or without arrhythmia, we found that the arrhythmia group was significantly younger (47.5 ± 4.6 vs. 51.6 ± 7.8 years, *p* = 0.02). Tricuspid atresia was the most common underlying CHD (*n* = 17, 58.6%) and atrio-pulmonary connection the most common Fontan type (*n* = 15, 51.7%). More details are provided in Table 1. 

**Figure 1:**
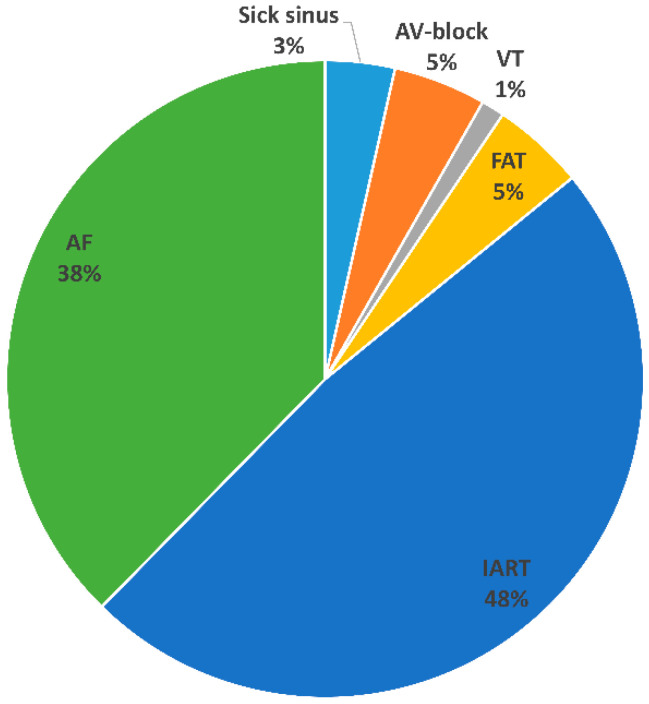
Arrhythmia type in SVP patients over 40 years.

Altogether, 85 arrhythmia episodes occurred in 29 patients. In 20 patients, a previous history of arrhythmia was recorded. The remaining nine patients developed arrhythmias “de novo” after their 40th birthday. Most common types were IART (48.2%) and AF (37.6%). More information is presented in Figure 1.

### 3.1. Electrophysiological Study with Ablation

At the begin of follow-up, 14 patients (48.2%) had already undergone 24 EPS (17 for IART, four for FAT, one for AF and two diagnostic EP studies). During follow-up, nine patients (32.1%, mean age 44 ± 4.2 years) underwent 18 EP procedures (range 1–4). Most common indication was IART (15 EPS (83.0%) in eight patients). In seven procedures, more than one IART type was found. Furthermore, five patients (55%) had two or more EPS. Time to arrhythmia recurrence after EPS was median 10.5 months (IQR 8–49 months). Immediate procedural success was achieved in 72.2% of cases (Table 2). During follow-up, six out of nine patients had arrhythmia recurrence requiring treatment by EPS or DCCV.

### 3.2. Direct Current Cardioversion

Before reaching the age of 40, 23 DCCV had been already performed in 12 patients (16 for IART and seven for AF). A total of 52 DCCV were performed in 20 patients (68.9%, mean age 43.4 ± 3.6 years) during follow-up. DCCV was performed for AF (27 DCCV (51.9%) in 11 patients), for IART (21 DCCV (40.3%) in 12 patients) or for FAT (three DCCV (5.7%) in two patients and VT in one patient (1.9%; Table 3). Altogether, 14 patients (70%) received more than one DCCV. Time to arrhythmia recurrence after DCCV was median 8 months (IQR 1.5–18.5). Initial conversion into sinus rhythm was achieved in 98% of patients.

### 3.3. Combined Intervention (EPS and DCCV)

Eight out of nine patients with EPS (88.9%) had one or more DCCVs: before (five patients) or after ablation (three patients). Time of DCCV after ablation was median 30 months (IQR 8–35 months). 

### 3.4. Medical Therapy

Amiodarone therapy as the only treatment was used in one patient (age 41 years). This patient had tricuspid atresia palliated with shunts. Amiodarone was initiated due to AF. At the last ECG, sinus rhythm was present. All other patients treated with amiodarone (*n* = 16, 55.2%), had additionally at least one EPS or DCCV. 

### 3.5. Device Therapy

In seven patients (24.1%), a pacemaker implantation was performed for the first time after the age of 40 years. Reasons for pacemaker implantation were higher degree AV-block (four patients, 57.1%) or sinus node dysfunction (three patients, 42.8%). Epicardial pacemaker leads were used in five patients (71.4%) and endocardial pacemaker leads in two patients (28.5%). An ICD with epicardial leads was implanted in one patient for secondary prophylaxis after a sustained ventricular tachycardia. Arrhythmias were detected in six/eight patients (75%) with device therapy during follow-up. Most common were IART (57%) and AF (31.4%). 

### 3.6. Outcomes

During follow-up, eight patients with arrhythmia (27.5%) died. This was significantly higher than in the group without arrhythmias (two patients, *p* = 0.007). Cause of death in patients with arrhythmias was cardiac in six patients (75%: one valve thrombosis, two heart failure, three sudden deaths) and non-cardiac in two patients (25%: one liver cirrhosis and one sepsis secondary to pneumonia). 

During the follow-up period, one patient underwent a Fontan conversion surgery at the age of 49 years due to a failing Fontan circulation and multiple atrial arrhythmias. This patient died one year after the operation. No conversions or heart transplants occurred in the group without arrhythmias. 

Arrhythmia recurrence occurred in 19/29 patients (65.5 %). Patients with more than one procedure had significantly more often PAH (6 vs. 0 patients; *p* = 0.046). On univariate Cox regression analyses, older age (HR 0.57, 95% C.I. 0.43–0.76, *p* < 0.0001) and severely impaired ventricular function (HR 17.03, 95% C.I. 1.54–188.5, *p* = 0.02) were predictors of recurrence. On multivariate analysis, only older age remained as an independent predictor (HR 0.58, 95% C.I. 0.43–0.78, *p* < 0.0001) (Table 4).

## 4. Discussion

The main findings of this retrospective study of patients with SVP older than 40 years with arrhythmias was that patients suffered mostly from IART and AF. Interestingly, chronic antiarrhythmic medication was rarely used as a treatment strategy for AT. Patients were mainly treated with DCCV and EPS/ablation. However, acute ablation success was limited to 72%. More than 65% of patients suffered from arrhythmia recurrence during follow-up; only 30% of patients receiving DCCV were arrhythmia-free at follow-up. Older age was found as an independent risk factor for arrhythmia recurrence. 

In patients with single ventricle physiology, ATs are a common complication and more than half of patients will develop rhythm disturbances 5 to 10 years after Fontan palliation [9]. Patients who develop AT are more prone to have atrial thrombi, enlarged right and left atria, heart failure, ventricular dysfunction and hospitalizations [1,10]. Moreover, a 6-year mortality risk of more than 40% has been described in patients with atrio-pulmonary shunts and atrial arrhythmias [1], as well as an association with Fontan failure [11]. The prevalence of AT in pediatric patients with Fontan circulation has been reported as 16–41%, even though contemporary cohorts describe a prevalence of around 9.4% [12]. The most frequent AT at this younger age group is cavo-tricuspid (CTI) and non-CTI related intra-atrial reentrant tachycardia (IART) [9,12]. Even though IART is the most common arrhythmia in almost 70% of complex CHD, the incidence of atrial fibrillation also increases with age [13]. In our study, we found AT in almost 60% of our patients over the age of 40 years (almost 50% IART and 40% AF). Compared to younger patients with SVP in whom the most common arrhythmia is IART [9,12], a remarkable increase of AF has been seen in this study. However, as pointed out our patients were much older than those described in previous studies, and additionally an atrio-pulmonary Fontan or no Fontan palliation at all was more common in our cohort in comparison [12]. AF can be triggered by scar tissue in the atria [13,14], chronic atrial pressure or volume overload and foci outside the pulmonary veins (e.g., right atrium or superior vena cava) [14]. Moreover, other factors associated with older age, such as higher body mass index, arterial hypertension, dyslipidemia, cigarette smoking and coronary artery disease, might favor the presence of AF [14]. According to our results, 90% of our patients with IART/AF had previous cardiac surgeries, most frequently atrio-pulmonary Fontan (55%) and TCPC (20%). Additionally, atrial enlargement and chronic volume and/or pressure overload are substrates for AF [14]. The presence of AF in patients with Fontan circulation has also been associated with sudden cardiac death [15].

Acute procedural success of EP in SVP was achieved in around 71–72% of cases in different studies [16,17]. These retrospective studies included all adult patients with simple to complex CHD, who had undergone an EP during an observation period of more than 10 years. Mean age was 32 ± 15 years (range, 1.3–70 years) [16] and 29 ± 14 years [17], respectively. The number of patients with single ventricle/Fontan circulation ranged between 14 and 21% [16,17]. Despite the differences in type of patients and age between these studies and ours, we also achieved an initial success rate of around 72%. In non-Fontan CHD, acute success has been reported in 85% of the procedures [16]. Acute procedural success in ablation therapy has been reported as a predictor of recurrence-free survival [17]. In our study, recurrence of AT after EP was seen in 55% of our patients. Among 20 patients with Fontan circulation and EP included in Grubb and colleagues’ study, 65% had a recurrence of AT, compared to 40% of non-Fontan patients [17]. Furthermore, median time to recurrence was 12.5 months in Fontan patients, compared to 50.1 months in other CHD patients (*p* = 0.01). In our cohort, median time to recurrence was 8 months. 

DCCV was used quite often for acute tachyarrhythmia treatment. It is the therapy of choice in acute unstable AT and VT [15]. EPS in complex CHD (like SVP) should be performed preferably in experienced centers [15]. While all EPS were performed in our center, DCCVs were not always performed at our hospital, as patients with acute symptoms usually present to their nearest hospital.

DCCV was the treatment of choice in 52 tachyarrhythmia episodes, of which more than 50% were AF and 40% IART. Acute success rate was high with 98%. However, not unexpected, 70% of patients had arrhythmia recurrence with a median time until recurrence of only 2.5 months. In a previous study with 279 adult patients with any kind of CHD (mean age of 55 ± 20 years, 22% with Fontan circulation), DCCV was successful in 94% of cases and recurrence was reported in 83% of cases [18]. After one year, only 40% remained in sinus rhythm [18]. Fontan circulation was a risk factor for DCCV failure and, together with AF, a risk factor for arrhythmia recurrence [18]. Medical antiarrhythmic treatment might be temporarily helpful for the prevention of arrhythmias after DCCV. However, in patients with SVP, which mainly are treated with amiodarone, recurrence despite medication has been described [1,2].

On the whole CHD spectrum, only 50% of patients remain arrhythmia free after catheter ablation in non-CTI dependent IART [15]. In patients with Fontan circulation, IART is often non-CTI dependent. It progresses along the lateral atrial wall and near the inferior vena cava [15]. In our study, arrhythmia recurrence was found in 19 patients (65.5%) and the multivariate Cox regression analyses identified older age as a risk factor for recurrence. Older age has already been associated with higher risk of arrhythmia. In complex CHD, 50% of those who reach the adulthood will develop an arrhythmia by age 65 years [19]. Other risk factors for recurrence that have been previously described are Fontan circulation and elevated BMI [20]. The finding that on one hand patients with arrhythmia are significantly younger than patients without arrhythmia, but on the other, older age was a risk factor for arrhythmia recurrence, merits further discussion. We believe that, while patients without arrhythmia have a better chance of survival to older age, once a patient develops arrhythmias, older age is a risk factor for recurrences due to the already described factors.

Regarding bradyarrhythmias, 24% of patients needed a pacemaker implantation during follow-up in our study. Sinus node dysfunction, higher-degree AV block, prolongation of atrial refractoriness and delayed atrial conduction have been described in Fontan patients [21]. Additionally, bradyarrhythmias can be associated with IART as a consequence of loss of atrioventricular synchrony and sinus node dysfunction [13]. According to our results, 75% of our patients with a new device implantation had had arrhythmias during the follow-up. IART was the most common arrhythmia in 60% of the cases. In younger cohorts, mostly after TCPC, pacemaker implantation has been reported in 13% of cases [1].

Sustained ventricular tachycardia leading to secondary ICD implantation occurred in one patient in our study. Ventricular arrhythmias are less frequent compared to AT in patients with SVP [1,12]. The presence of scar tissue at ventricular level might be a risk factor for ventricular tachyarrhythmias. On the other hand, sudden death was the cause of death in three patients, which could have an arrhythmic origin [22]. Sudden deaths have been reported in 9–17% in late Fontan [9] and the annual incidence of sudden death is about 0.15%/year, comparable to other pathologies such as Tetralogy of Fallot and aortic coarctation. However, it is still not clear which patients would benefit from an ICD for primary prevention.

### Limitations

The retrospective nature of the study is inevitably associated with missing or limited information. We describe a cohort of SVP patients over the age of 40 years and therefore around 20% of patients were unoperated and another 20% were only palliated (e.g., with shunts). Additionally, age at first Fontan was much older than at the present time. Furthermore, the large majority (92%) had a left ventricular morphology. The next generation of these patients will most likely be different due to different surgical techniques (TCPC) and timing of surgery. This will affect type and frequency of arrhythmias.

## 5. Conclusions

In conclusion, patients with SVP over the age of 40 years are at high risk of developing arrhythmias, especially IART and AF. Arrhythmia complexity increases with age, AF being one of the most prevalent tachyarrhythmia in this age group. DCCV treatment is quite effective in the acute phase. EPS should be offered to patients at experienced centers, but recurrences are common and treatment remains challenging.

## Figures and Tables

**Table 1:** Baseline characteristics.

	All	with Arrhythmias	without Arrhythmias	*p*
*n* (%)	49 (100.0)	29 (59.2)	20 (40.8)	
Age (years)	49.2 ± 6.4	47.5 ± 4.6	51.6 ± 7.8	0.02
Female, *n* (%)	19 (38.8)	11 (37.9)	8 (40)	0.88
Congenital heart defect, *n* (%)				
Double-inlet left ventricle	23 (46.9)	9 (31)	14 (70)	0.007
Tricuspid atresia	20 (40.8)	17 (58.6)	3 (15)	0.002
Miscellaneous ‡	6 (12.2)	3 (10.3)	3 (15)	0.62
Type of cardiac surgery, *n* (%)				
Atrio-pulmonary connection	20 (40.8)	15 (51.7)	5 (25)	0.06
TCPC	11 (22.4)	7 (24.1)	4 (20)	0.8
Other surgical intervention †	9 (18.4)	4 (13.8)	5 (25)	0.32
No surgical interventions (native)	9 (18.4)	3 (10.3) *	6 (30) **	0.08
Age at first Fontan (years)	45 (91.8)	16.3 ± 7.1	24.3 ± 10.9	0.02
Ventricle morphology, *n* (%)				0.7
Left	45 (91.8)	27 (93.1)	18 (90)	
Right	4 (8.1)	2 (6.9)	2 (10)	
Cyanosis, *n* (%)	27 (55.1)	14 (48.3)	13 (65)	0.25
PHVD	8 (16.3)	6 (19.3)	2 (10)	0.32
Cardiac medication, *n* (%)				
ACE-inhibitors/AT-Blocker	8 (16.3)	5 (17.2)	3 (15)	0.84
Beta-blocker	34 (69.4)	24 (82.8)	10 (50)	0.01
MRA	30 (61.2)	21 (72.4)	9 (45)	0.06
Digoxin	14 (28.6)	8 (27.6)	6 (30)	0.85
Amiodarone	17 (58.6)	17 (58.6)	0	<0.0001
Diuretics	36 (73.4)	23 (79.3)	13 (65)	0.27
Anticoagulation, *n* (%)				0.03
None	10 (20.4)	2 (6.9)	8 (40)	
Vitamin K-Antagonist	36 (73.5)	25 (86.2)	11 (55)	
Direct oral anticoagulants	1 (2.0)	1 (3.4)	0	
ASA	1 (2.0)	0	1 (5)	
ASA + Clopidogrel	1 (2.0)	1 (3.4)	0	
Advanced PAH therapies, *n* (%)	8 (16.3)	4 (13.8)	4 (20)	0.47
NYHA class, *n* (%)				0.25
I	11 (22.4)	5 (17.2)	6 (30)	
II	29 (59.2)	20 (68.9)	9 (45)	
III	9 (18.4)	4 (13.8)	5 (25)	
Ventricular function, *n* (%)				
Normal	11 (22.4)	6 (20.7)	5 (25)	0.72
Mild	17 (34.7)	8 (27.6)	9 (45)	0.2
Moderate	17 (34.6)	11 (37.9)	6 (30)	0.57
Severe	4 (8.2)	4 (13.8)	0	0.08

ASA: acetylsalicylic acid; MRA: mineralocorticoid-receptor-antagonist; NYHA: New York Heart Association class; PHVD: pulmonary hypertensive vascular disease. † Other surgical intervention: shunts, AV-valve repair or conduit implantation. ‡ Miscellaneous include: criss-cross heart, pulmonary + tricuspid atresia, single ventricle. * Tricuspid atresia and large intra-atrial septal defect (3 patients). ** Double inlet left ventricle (4 patients), tricuspid atresia and large intra-atrial septal defect (1 patient), miscellaneous (1 patient).

**Table 2:** Patient characteristics, arrhythmia type, EP procedure and success.

Patient No.	CHD	Type of Surgery	Sex	Type of Arrhythmia	Procedure	Acute Success	Complications/Anatomical Features
1	TA	native CHD	female	AF	Isolation PVs; cavo-pulmonary isthmus ablation, left atrial lines	yes	no
				AF	Re-isolation PV	yes	no
				AF	Re-isolation PVs	yes	no
2	TA	Atrio-pulmonary connection	female	IART	CT isthmus ablation; ablation from tricuspid ring to scar tissue in the RA-PA connection	yes	no
				IART + FAT	Ablation IART around tricuspid valve; overdrive pacing of FAT in PA zone	yes	no
3	DILV	Atrio-pulmonary connection	male	typical atrial flutter + IART	ablation CT isthmus; ablation IART at the atrial septum	yes	no
4	single ventricle	Atrio-pulmonary connection	female	IART + AF	ablation line from CT isthmus to left inferior PV; Isolation of PVs;	no	no/azygos continuation
				IART	ablation line from CT isthmus to left inferior PV	yes	no/azygos continuation
5	TA	Atrio-pulmonary connection	male	1. IART 2. IART	1. ablation around RA-PA conduit 2. ablation line between conduit and lateral atriotomy	no	no
				multiple IARTs	mechanical termination of IART; ablation at anterolateral conduit side	no	no
				IART	ablation around RA-RV conduit and lateral RA side	yes	no
				IART (2 forms)	1. septal ablation, cranio-posterior side of RA-RV conduit 2. ablation around right PVs	no	no/retrograde access (aorta) for PVs ablation
6	TA	Atrio-pulmonary connection	female	IART	ablation at CS ostium	yes	no
7	TA	Atrio-pulmonary connection	male	IART	ablation antero-inferior RA	yes	no
8	criss-cross heart	Shunts	male	multiple IARTs (3 different)	1. Ablation in anterior-cranial zone of Björk tunnel 2. Ablation in posterior- cranial zone of Björk tunnel 3. IART not ablatable, ECV	no	no
				IART	widened ablation in RA	yes	hypotension requiring noradrenalin and O2 supply
9	TA	TCPC	male	unstable IART	CT isthmus ablation; lateral line ablation around RA scar	yes	no
				unstable IART	ablation in posterior RA, at transition with SVC	yes	no

AF: atrial fibrillation; CT: cavo-tricuspid; CS: coronary-sinus; DILV: double inlet left ventricle; ECV: electrical cardioversion; FAT: focal atrial tachycardia; IART: intra-atrial reentrant tachycardia; PV: pulmonary vein; RA: right atrium; SVC: superior vena cava; TA: tricuspid atresia.

**Table 3:** Underlying arrhythmia necessitating DCCV during follow-up.

Patients.	Type of Surgery			Arrhythmias		
		Atrial fibrillation	IART	Focal atrial tachycardia	Ventricular tachycardia	Total number shocks/patient
Patient 1	Native	4				4
Patient 2	Native	3				3
Patient 3	TCPC conversion	3				3
Patient 4	TCPC conversion		1			1
Patient 5	TCPC directly		1			1
Patient 6	TCPC directly		3			3
Patient 7	TCPC directly	3			1	4
Patient 8	Atrio-pulmonary connection		1			1
Patient 9	Atrio-pulmonary connection		2			2
Patient 10	Atrio-pulmonary connection	2				2
Patient 11	Atrio-pulmonary connection	2	3	1		6
Patient 12	Atrio-pulmonary connection	2				2
Patient 13	Atrio-pulmonary connection	1				1
Patient 14	Atrio-pulmonary connection	3	1	2		6
Patient 15	Atrio-pulmonary connection		2			2
Patient 16	Atrio-pulmonary connection		3			3
Patient 17	Atrio-pulmonary connection		1			1
Patient 18	Atrio-pulmonary connection	1	2			3
Patient 19	Shunts		1			1
Patient 20	Shunts	3				3

**Table 4:** Risk factors for arrhythmia recurrence.

	Univariate HR (95% C.I.)	*p*	Multivariate HR (95% C.I.)	*p*
Age	0.57 (0.43–0.76)	<0.0001	0.58 (0.43–0.78)	<0.0001
Age at first Fontan	0.93 (0.84–1.02)	0.13		
Sex	1.03 (0.39–2.72)	0.95		
Type CHD	1.29 (0.63–2.63)	0.49		
Cirrhosis	1.7 (0.55–5.48)	0.34		
Impaired renal function	0.99 (0.38–2.62)	0.98		
Atrio-pulmonary connection	1.75 (0.63–4.88)	0.28		
Native CHD	0.33 (0.043–2.55)	0.29		
Severely impaired ventricular function	17.03 (1.54–188.5)	0.021	8.57 (0.75–97.82)	0.084
PHVD	1.33 (0.47–3.77)	0.59		
Cyanosis	0.68 (0.26–1.83)	0.45		
Arrhythmia before age 40	1.54 (0.38–6.3)	0.55		

CHD: congenital heart disease; PHVD: pulmonary hypertensive vascular disease.

## Data Availability

The data underlying this article cannot be shared publicly due to data privacy reasons and the according German regulations.
